# Spatiotemporal dynamics and environmental determinants of scrub typhus in Anhui Province, China, 2010–2020

**DOI:** 10.1038/s41598-023-29373-7

**Published:** 2023-02-06

**Authors:** Xianyu Wei, Junyu He, Wenwu Yin, Ricardo J. Soares Magalhaes, Yanding Wang, Yuanyong Xu, Liang Wen, Yehuan Sun, Wenyi Zhang, Hailong Sun

**Affiliations:** 1 Department of Epidemiology and Biostatistics, School of Public Health, Anhui Medical University, Hefei, China; 2 Chinese PLA Center for Disease Control and Prevention, Beijing, China; 3 Ocean College, Zhejiang University, Zhoushan, China; 4 Ocean Academy, Zhejiang University, Zhoushan, China; 5 Chinese Center for Disease Control and Prevention, Beijing, China; 6 Spatial Epidemiology Laboratory, School of Veterinary Science, The University of Queensland, Brisbane, Australia; 7 Child Health Research Center, The University of Queensland, Brisbane, Australia

**Keywords:** Risk factors, Health policy, Public health

## Abstract

This study aims to describe the epidemiological characteristics of scrub typhus, detect the spatio-temporal patterns of scrub typhus at county level, and explore the associations between the environmental variables and scrub typhus cases in Anhui Province. Time-series analysis, spatial autocorrelation analysis, and space–time scan statistics were used to explore the characteristics and spatiotemporal patterns of the scrub typhus in Anhui Province. Negative binomial regression analysis was used to explore the association between scrub typhus and environmental variables. A total of 16,568 clinically diagnosed and laboratory-confirmed cases were reported from 104 counties of 16 prefecture-level cities. The number of female cases was higher than male cases, with a proportion of 1.32:1. And the proportion of cases over 65 years old was the highest, accounting for 33.8% of the total cases. Two primary and five secondary high-risk clusters were detected in the northwestern, northeastern, and central-eastern parts of Anhui Province. The number of cases in primary and secondary high-risk clusters accounted for 60.27% and 3.00%, respectively. Scrub typhus incidence in Anhui Province was positively correlated with the population density, normalized difference vegetation index, and several meteorological variables. The mean monthly sunshine duration with 3 lags (SSD_lag3), mean monthly ground surface temperature with 1 lag (GST_lag1), and mean monthly relative humidity with 3 lags (RHU_lag3) had the most significant association with increased cases of scrub typhus. Our findings indicate that public health interventions need to be focused on the elderly farmers in north of the Huai River in Anhui Province.

## Introduction

Scrub typhus is a vector-borne zoonotic disease transmitted to humans through the bite of the larvae of trombiculid mites. The causative bacterium of this disease is *Orientia tsutsugamushi* (*O. tsutsugamushi*)^1^. The illness can be characterized by rash, eschar, or ulcer at the bite site, and its clinical manifestations exhibit a wild variety from mild fever to fatal multi-organ dysfunction^2^. The traditional endemic area of scrub typhus named ‘Tsutsugamushi Triangle’ includes more than half population of the world and covers most parts of the Asia–Pacific region^3^. However, recent studies suggested that scrub typhus endemic areas are no longer confined to the ‘Tsutsugamushi Triangle’^4^, and the number of cases has been increased in the traditional epidemic regions^5^.

Scrub typhus has become prevalent again during recent decades^1^. In China, not only the spatial distribution of scrub typhus has been expanding to the north of the Yangtze River since the late 1990s, but also the incidence of scrub typhus has been increasing rapidly over the past decade^5–7^. Anhui Province is a severe epidemic region of scrub typhus in the north of the Yangtze River. The cases of scrub typhus have been reported in Anhui Province every year since the outbreak in 2008^8^. However, the characteristics of scrub typhus epidemics in Anhui Province are unclear and their spatiotemporal patterns need to be investigated. It is necessary to expand our knowledge of the epidemiological characteristics, spatio-temporal patterns of this reemerging disease, as well as the associations between the environmental variables and scrub typhus in Anhui Province. A detailed understanding of scrub typhus in Anhui Province will provide scientific basis for targeted interventions against this disease.

## Materials and methods

### Study area

**Figure 1:**
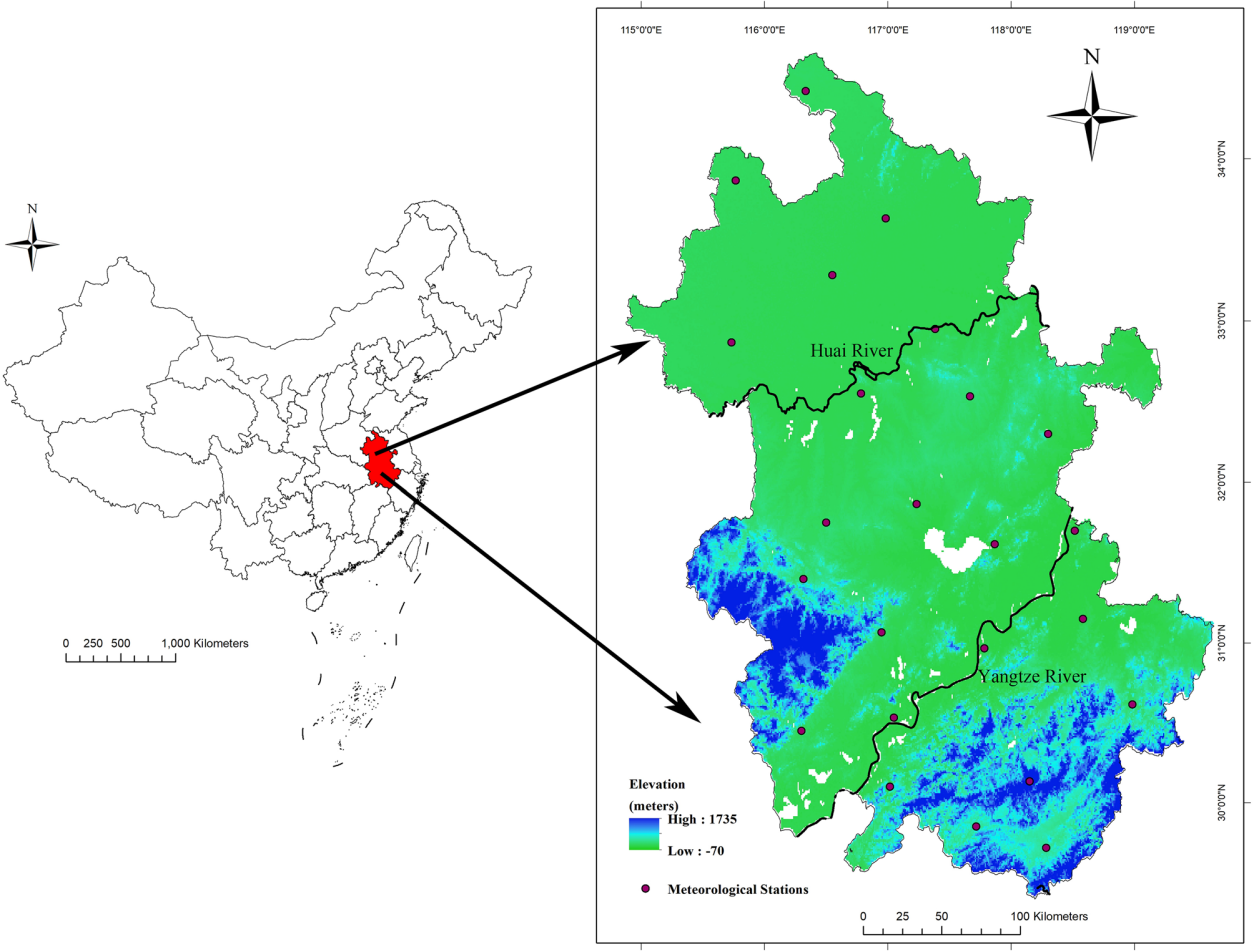
The geographic location and the color altitude map of Anhui province. Geographical distribution of 24 meteorological stations (purple dots) where meteorological data were acquired for this study. The base layer of the map was obtained from Resource and Environment Science and Data Center (https://www.resdc.cn/Datalist1.aspx?FieldTyepID=7,1).

Anhui Province (29°41′–34°38′ N, 114°54′–119°37′ E) is located in the Yangtze River Delta, Eastern China (Fig. 1). It is bordered with Jiangsu Province in the east, the provinces of Henan and Hubei in the west, Zhejiang Province in the southeast, Jiangxi Province in the south and Shandong Province in the north. It covers approximately 140,100 km^2^, with 16 prefecture-level cities and 104 counties. The population reached 61.03 million people in 2020, with a population density of 436 people/km^2^. The Huai River locates in the temperate and subtropical transition zone, traverses Anhui Province, and there is a warm temperate semi-humid monsoon climate to the north of the Huai River and a subtropical humid monsoon climate to the south of the Huai River. The annual mean temperature is between 14 and 17 ℃, the annual average precipitation ranges from 773 to 1670 mm and the per capita gross domestic product is 58.49 thousand Chinese Yuan (CNY) (Statistics Bureau of Anhui Province, 2019).

### Ethics statement

This study was approved by the Ethics Committee of the Chinese PLA Center for Disease Control and Prevention and the Chinese Center for Disease Control and Prevention. All the data analyzed in this study were de-identified to protect patient confidentiality. And the aggregate data were used to analyze in our study. All methods were carried out in accordance with relevant guidelines and regulations.

### Data collection and management

In China, all clinically diagnosed and laboratory-confirmed cases of scrub typhus are required by law to be reported to the Chinese Center for Disease Control and Prevention (China CDC). All individual cases of scrub typhus were diagnosed based on the diagnosis criteria and case classification guidelines issued by China CDC (Appendix Table 1https://www.chinacdc.cn/tzgg/200901/t20090105_40316.html). The scrub typhus data in Anhui Province were collected from China CDC.

The county-level map at the 1:1,000,000 scale of Anhui Province was collected from the Data Center for Geographic Sciences and Natural Resources Research, CAS (http://www.resdc.cn/). The annual resident demographic data were obtained from the Anhui Provincial Bureau of Statistics (http://tjj.ah.gov.cn/ssah/qwfbjd/tjnj/index.html). Monthly meteorological variables including monthly air temperature (TEM) (℃), mean monthly evaporation (EVP) (mm), mean monthly wind speed (WIN) (m/s), mean monthly ground surface temperature (GST) (℃), monthly cumulative precipitation (PRE) (dm), mean monthly pressure (PRS) (hPa), mean monthly relative humidity (RHU) (%) and mean monthly sunshine duration (SSD) (h) were obtained from China Meteorological Data Service Center (http://data.cma.cn). A total of 24 meteorological stations are distributed in Anhui Province (Marked with red dots in Fig. 1). Kriging interpolation and Zonal Statistics in ArcGIS software (version 10.6, ESRI Inc., Redlands, CA, USA) were used to calculate averaged values of each meteorological variable for each county. Elevation and Slop TIFF files with a spatial resolution of 1 × 1 km^2^ were obtained from the Global Digital Elevation Data Products (http://www.gscloud.cn). The normalized difference vegetation index (NDVI) data sets with a 1 × 1 km^2^ spatial resolution and the raster data set of GDP during 2010–2020 were downloaded from Resource and Environment Science and Data Center (https://www.resdc.cn/data.aspx?DATAID=343).

### Epidemiological features analysis

To describe the epidemiological characteristics and quantify differences in the annual incidence by gender, age group, and occupation of scrub typhus in Anhui Province, the annual incidence rates were tabulated and a chi-square (χ^2^) test was conducted with SPSS software (version 23, IBM Corp, Armonk, NY, USA). A heat map of monthly incidence was created over 132 months to determine whether seasonal trends and temporal dynamics were consistent across prefecture-level cities. Temporal autocorrelation between the monthly notified cases of scrub typhus was conducted by calculating the autocorrelation function (ACF) to detect the presence of autocorrelation for time lags between 0 and 80 months. In addition, a series of incidence maps at the county level was created to display the change in spatial distribution.

### Spatial autocorrelation analysis

Firstly, Global Moran’s *I* was adopted to explore the average correlation among all counties in Anhui Province. The Global Moran’s *I* can be expressed as follows:

$$I=\frac{n\sum_{i=1}^{n}\sum_{j=1}^{n}{\omega }_{ij}({x}_{i}-\mu )({x}_{j}-\mu )}{\sum_{i=1}^{n}\sum_{j=1}^{n}{\omega }_{ij}\sum_{i=1}^{n}{({x}_{i}-\mu )}^{2}},$$

where* I* denotes Moran’s* I* *index*; *x*_*i*_ is the annual incidence of the* i*th county in the Anhui Province; *μ* represents the mean of *x*_*i*_; n equates to the total number of counties; *ω*_*ij*_ refers to the spatial weight between county *i* and *j* in the spatial contiguity^9^. The closer Moran’s* I* gets to 1, the higher aggregated the whole incidence is (i.e., high-value aggregation or low-value aggregation). The closer it gets to − 1, the more dispersed the whole incidence is. The spatial distribution of incidence may be the result of random spatial processes if the value of Moran’s* I* is around 0^5,7^. To evaluate the significance of that index, we performed the Z-test to calculate both a z-score and *p*-value. The z-score is computed as

$$Z=\frac{I-E(I)}{\sqrt{var(I)}},$$

where $E\left(I\right)=-1/(n-1)$; $var\left(I\right)=E\left({I}^{2}\right)-E{(I)}^{2}$; When the returned *p*-value is less than 0.05, the null hypothesis can be rejected.

Secondly, Local Moran’s *I* for local indication of spatial autocorrelation (LISA) identifies spatial clusters of counties with high or low annual incidence and spatial outliers. The Local Moran’s *I* statistic is given as

$${I}_{i}=\frac{{x}_{i}-\overline{X} }{\sum_{j=1,j\ne i}^{n}{\left({x}_{i}-\overline{X }\right)}^{2}/(n-1)}\sum_{j=1,j\ne i}^{n}{\omega }_{ij}({x}_{j}-\overline{X }),$$

All the parameters here have the same meaning as before. Z-test was also performed to calculate the z-score which determines the significance level of clusters. A high positive z-score for a county indicates that the surrounding counties have similar incidences (either High-High or Low-Low). A low negative z-score for a county indicates if the county has a high value and is surrounded by counties with low incidence (High-Low) or if the county has a low incidence and is surrounded by counties with high incidence (Low–High)^10^.

### Spatial–temporal cluster analysis

Kulldorff’s space–time scan statistic was applied to detect space–time clusters for monthly cases of scrub typhus in Anhui Province at the county level based on a discrete Poisson model using the SaTScan software (version 9.3, https://www.satscan.org/). The space–time scan statistic is defined by a variable-sized cylindrical moving window with a circular geographic base which is in turn centered on each of several possible counties positioned throughout the study region and with height corresponding to time^11^. The null hypothesis is that there is no difference in relative risk (RR) of the incidence within and outside the window^12^. The Log Likelihood Ratio (*LLR*) was calculated by the following formula to assess the difference in the incidence inside and outside the window:

$$LLR = \log \left\{ {(c/n)^{c} \left[ {\frac{C - c}{{C - n}}} \right]^{(C - c)} } \right\}$$

where *C* equates to the total number of cases; *c* denotes the number of observed cases inside the window; *n* refers to the number of expected cases inside the window. The windows with an *LLR* value greater than 1000 were defined as the primary clusters and other windows with statistically significant *LLR* values were defined as secondary clusters. We performed the space–time scan with the maximum spatial cluster size setting at 10% of the total population and the maximum temporal size setting at one month. The number of Monte Carlo replications was set to 999 and a two-sided *p*-value < 0.05 was considered statistically significant^13^.

### Association between scrub typhus and the environmental factors

To examine the association between monthly scrub typhus cases and potential environmental factors at the county level, we aggregated the monthly cases and potential environmental factors into a panel dataset and then conducted panel negative binomial regression analyses. The negative binomial distribution is suitable for the analysis of overly dispersed data^14^. Through verification, we found that our data of scrub typhus was over-dispersed.

Pearson’s correlation analysis was conducted to assess the correlation between co-variables, and highly correlated variables with a threshold of Pearson correlation |r|> 0.7 were not entered in the model simultaneously^15–17^. Then univariate negative binomial regressions were applied to explore statistically significant variables that would be incorporated into a multivariate negative binomial regression. The variables in the multivariate model were further selected by using the stepwise regression method and the final multivariate regression model included only those variables that reached a two-sided *p*-value < 0.01. Given that the mean incubation period of scrub typhus in humans is 10–12 days^17,18^, and that the life cycle of a chigger is about 2–3 months^19,20^, the 0 to 3 months lags effects of each meteorological factor on monthly cases were also taken into account. The incidence rate ratio (IRR) estimated using the maximum likelihood method was used to show the impact of each variable. The analysis was conducted in STATA 17.0 software (StataCorp. 2021. Stata Statistical Software: Release 17. College Station, TX: StataCorp LLC).

## Results

### Epidemiological features analysis

**Figure 2:**
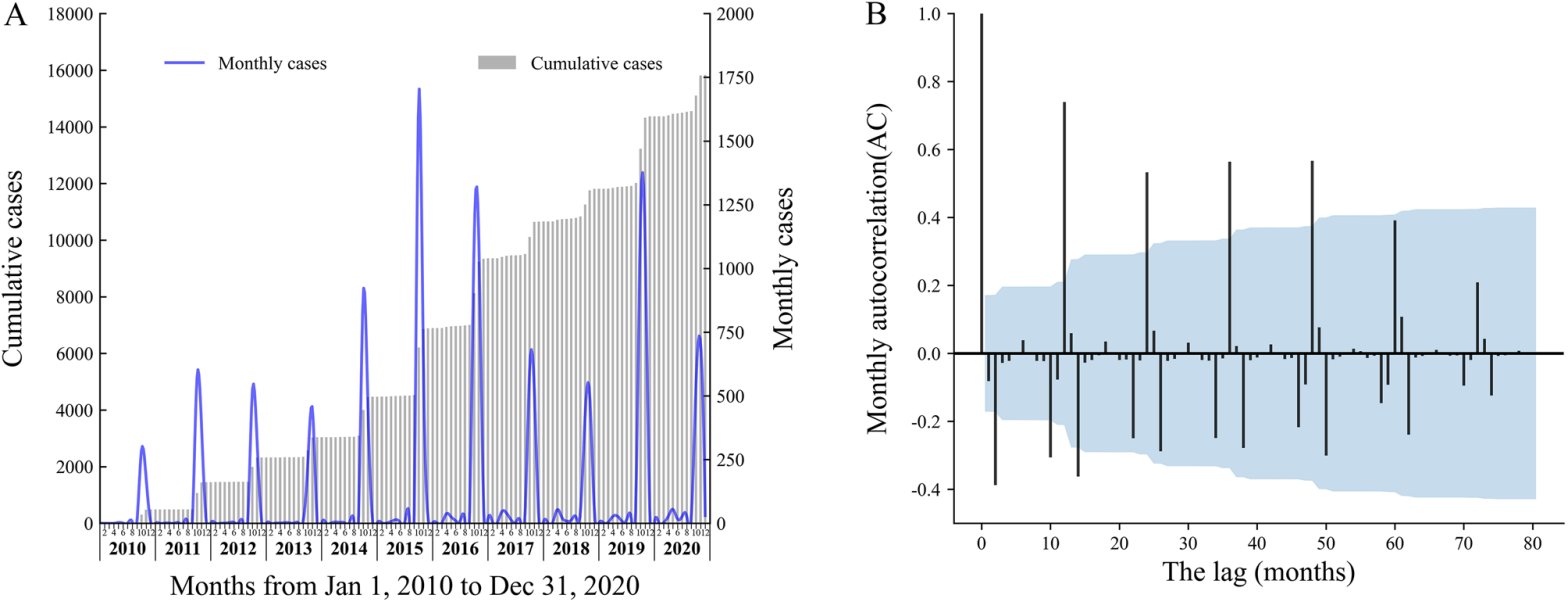
Temporal distribution and Seasonal patterns in reported scrub typhus cases from 2010 to 2020 in Anhui, China. The temporal distributions of reported scrub typhus cases in Anhui are presented as monthly number of cases (blue line) and cumulative cases (grey spikes) in panel A and the seasonal patterns as determined by monthly autocorrelation between the number of reported cases for each month is presented using time lags between 0 and 80 months in panel B.

**Figure 3:**
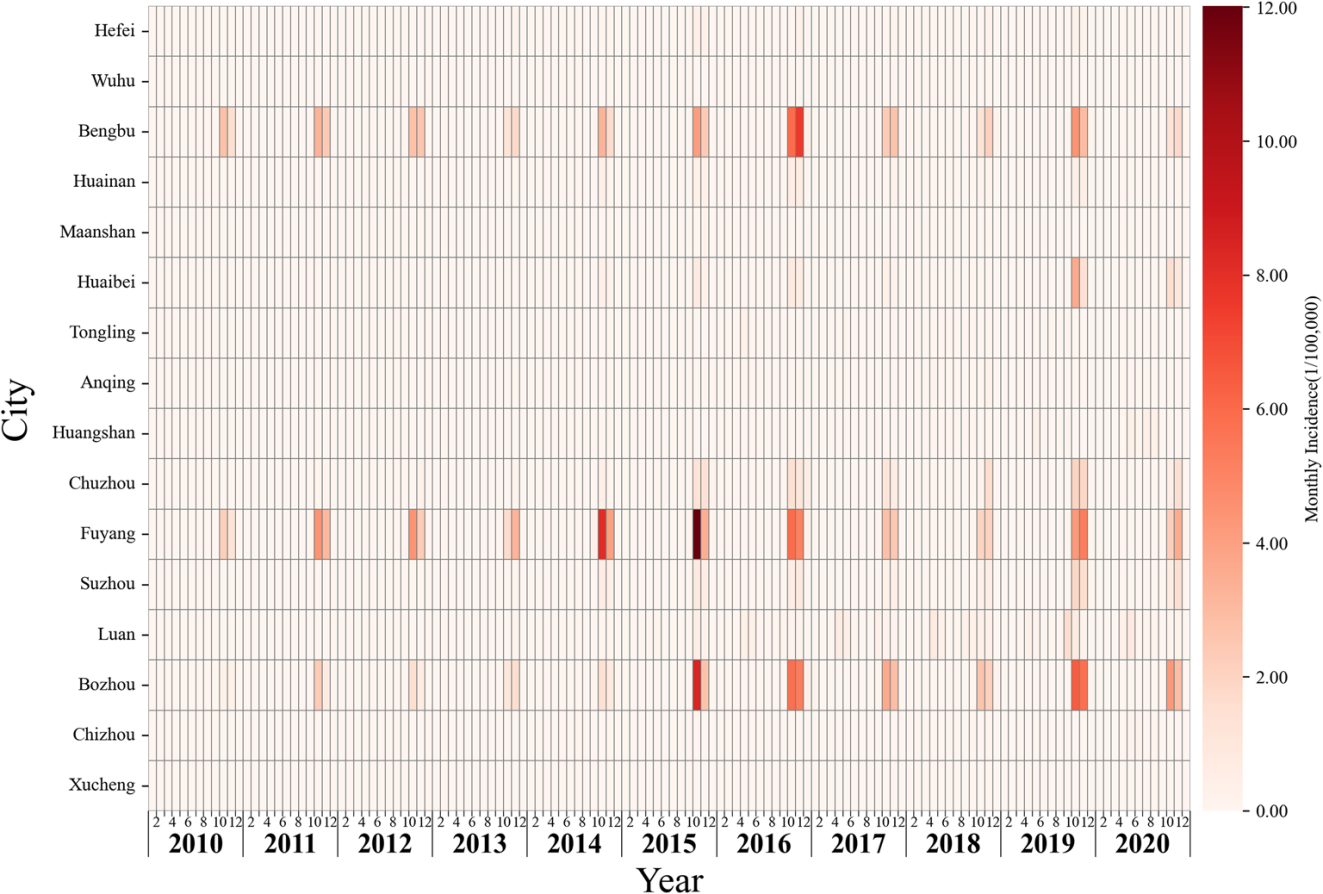
Heat map of monthly incidence of each city in Anhui, 2010–2020. Monthly incidence of all cities were shown in the heat map.

A total of 16,568 cases were reported in 104 counties from 16 prefecture-level cities in Anhui Province during 2010–2020. Among them, 9439 cases (56.97%) were female and 7129 cases (43.04%) were male. The differences between gender were statistically significant over the past 11 years (*p* < 0.05). The greatest number of occurred cases occurred in the group aged ≥ 65 years, followed by the age group 50–59 years. Farmers accounted for the highest proportion of scrub typhus in the occupation population with an increasing trend and there were also significant differences in the incidence among different age groups and occupations each year (Table 1). Overall, the occurrence of the disease was in a state of rhythmic fluctuation, with a mean annual incidence rate of 2.33 (95% Confidence Interval (CI): 1.56–3.10) per 100,000 population. Monthly changes in the number of cases showed that the epidemic peak regularly occurred in October–November with 87.64% of the total cases (Appendix Fig. 1). The highest number of monthly cases occurred in October 2015 (1681 cases), and the year with the largest number of cases was 2019 (2608 cases) (Fig. 2A). The pattern of autocorrelation was presented in Fig. 2B which demonstrated the maximum correlations every 12 months, and minimum correlations every 3 months. The heat map (Fig. 3) illustrated that the major high-incidence cities showed consistent seasonal trends and temporal dynamics.

**Table 1:** Annual number of scrub typhus cases and incidence rate by gender, age and occupation in Anhui province, China, 2010–2020.

Features		2010	2011	2012	2013	2014	2015	2016	2017	2018	2019	2020	Total	Mean annual incidence
Sex	Males	267 (0.88)	419 (1.39)	405 (1.35)	274 (0.90)	685 (2.23)	1079 (3.47)	1075 (3.44)	584 (1.85)	550 (1.73)	1144 (3.58)	647 (2.11)	7129	2.08
	Females	307 (1.05)	565 (1.91)	583 (1.96)	503 (1.68)	1079 (3.59)	1345 (4.44)	1396 (4.54)	739 (2.39)	609 (1.94)	1464 (4.62)	849 (2.80)	9439	2.81
	Pearson chi-square values (p-value)	4.28 (0.04)	24.24 (< 0.01)	33.61 (< 0.01)	70.79 (< 0.01)	97.44 (< 0.01)	36.59 (< 0.01)	47.19 (< 0.01)	21.46 (< 0.01)	4.02 (0.05)	42.39 (< 0.01)	29.24 (< 0.01)		
Age group	< 10	21 (0.28)	38 (0.51)	60 (0.80)	24 (0.31)	56 (0.71)	92 (1.19)	58 (0.74)	25 (0.32)	14 (0.18)	35 (0.44)	25 (0.33)	448	0.53
	10–19	20 (0.24)	22 (0.28)	30 (0.40)	15 (0.21)	31 (0.43)	49 (0.70)	31 (0.47)	23 (0.33)	32 (0.44)	25 (0.34)	23 (0.33)	301	0.38
	20–29	48 (0.56)	95 (1.28)	81 (1.10)	63 (0.81)	130 (1.44)	149 (1.60)	136 (1.49)	72 (0.84)	46 (0.59)	83 (1.19)	35 (0.52)	938	1.04
	30–39	52 (0.56)	76 (0.89)	78 (0.99)	51 (0.68)	117 (1.52)	177 (2.28)	151 (1.95)	96 (1.24)	72 (0.93)	123 (1.60)	82 (1.11)	1075	1.25
	40–49	115 (1.03)	181 (1.48)	164 (1.32)	119 (0.97)	256 (2.20)	396 (3.45)	377 (3.33)	236 (2.11)	164 (1.51)	341 (3.25)	157 (1.56)	2506	2.02
	50–59	114 (1.84)	191 (3.07)	188 (2.81)	122 (1.72)	314 (4.54)	462 (6.17)	502 (6.09)	285 (3.27)	278 (2.79)	666 (5.80)	436 (3.96)	3558	3.82
	60–64	62 (2.18)	129 (4.02)	125 (3.65)	139 (3.91)	312 (9.14.)	331 (9.66)	346 (9.65)	145 (4.01)	159 (4.68)	273 (9.57)	110 (4.02)	2131	5.86
	≥ 65	143 (2.35)	252 (3.70)	262 (3.62)	244 (3.31)	548 (7.69)	768 (10.66)	870 (11.70)	441 (5.70)	393 (4.79)	1062 (11.97)	628 (7.39)	5611	6.63
	Pearson chi-square values (p-value)	323.34 (< 0.01)	544.84 (< 0.01)	453.26 (< 0.01)	597.99 (< 0.01)	1503.10 (< 0.01)	1728.77 (< 0.01)	2136.90 (< 0.01)	906.85 (< 0.01)	896.14 (< 0.01)	2425.45 (< 0.01)	1432.45 (< 0.01)		
Farmers	Yes	491 (1.52)	860 (3.52)	834 (3.21)	690 (2.93)	1610 (7.19)	2114 (9.04)	2257 (10.04)	1202 (5.37)	1026 (5.56)	2397 (12.90)	1381 (7.76)	14,862	6.28
	No	83 (0.30)	124 (0.35)	154 (0.45)	87 (0.24)	154 (0.40)	310 (0.81)	214 (0.54)	121 (0.30)	133 (0.29)	211 (0.47)	115 (0.27)	1706	0.40
	Pearson chi-square values (p-value)	227.26 (< 0.01)	877.06 (< 0.01)	676.92 (< 0.01)	808.11 (< 0.01)	2248.23 (< 0.01)	2481.63 (< 0.01)	3242.06 (< 0.01)	1748.14 (< 0.01)	1977.53 (< 0.01)	4966.24 (< 0.01)	2886.93 (< 0.01)		
	Total	573 (0.82)	984 (1.62)	988 (1.46)	777 (1.18)	1764 (2.35)	2424 (3.93)	2471 (3.95)	1323 (2.10)	1159 (1.81)	2608 (4.00)	1496 (2.37)	16,568	2.33

**Figure 4:**
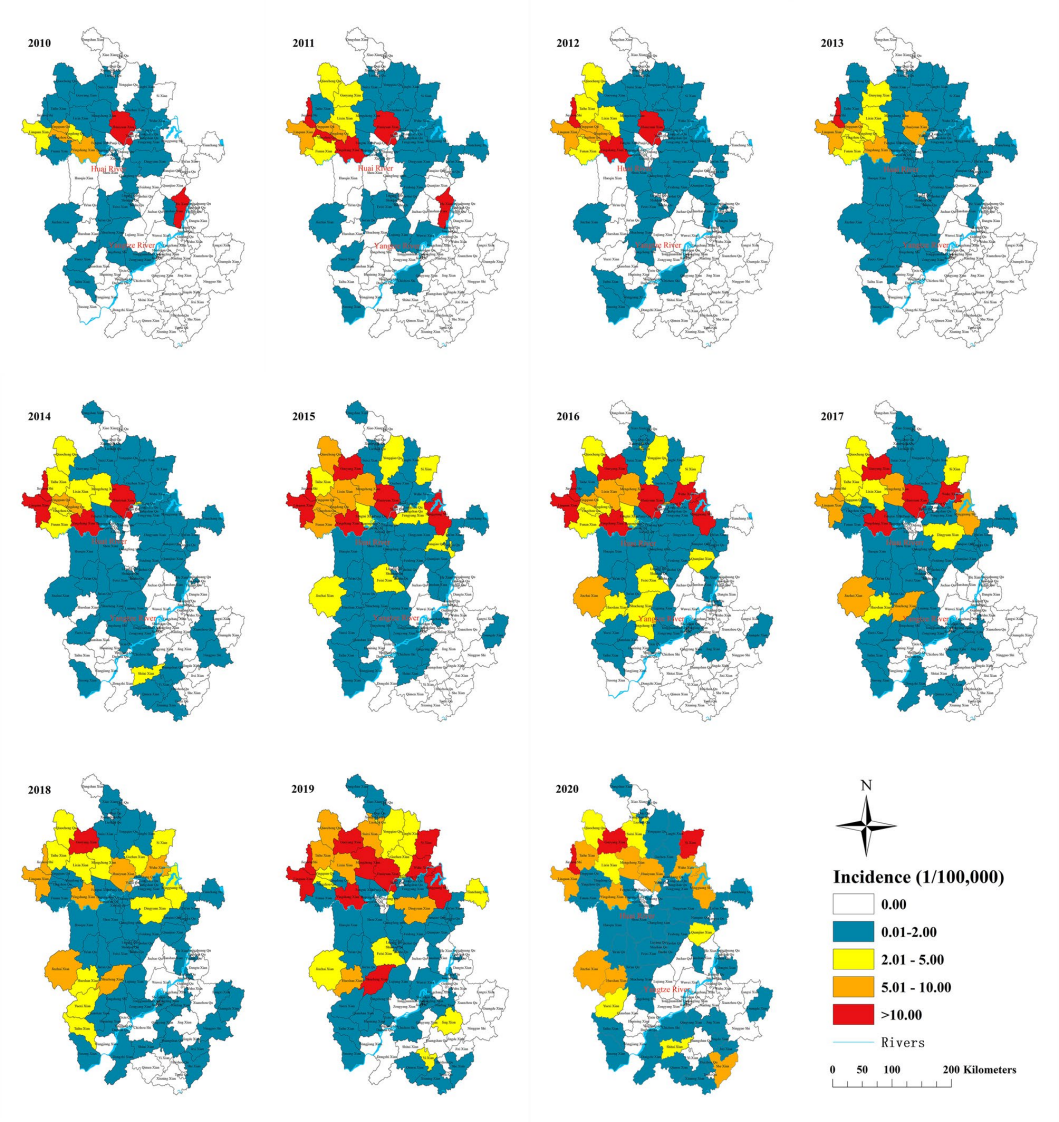
Spatial distributions in the annual incidence of scrub typhus in Anhui, China during 2010–2020. The base layer of the map was obtained from Resource and Environment Science and Data Center (https://www.resdc.cn/Datalist1.aspx?FieldTyepID=7,1).

The annual pattern of incidence in each county ranged from 0 cases to 41.88 cases per 100,000 residents. Counties with annual unreported cases have fallen from 76 in 2010 to 29 in 2020 (Fig. 4). The counties with high incidence were mainly concentrated in the north of the Huai River. For the past 11 years, the number of cumulative cases in the counties of Yingshang, Linquan, and Guoyang ranked in the top 3 and 85.91% of the cumulative cases occurred in the counties where located in the north of Huai River.

### Spatial autocorrelation analysis

**Figure 5:**
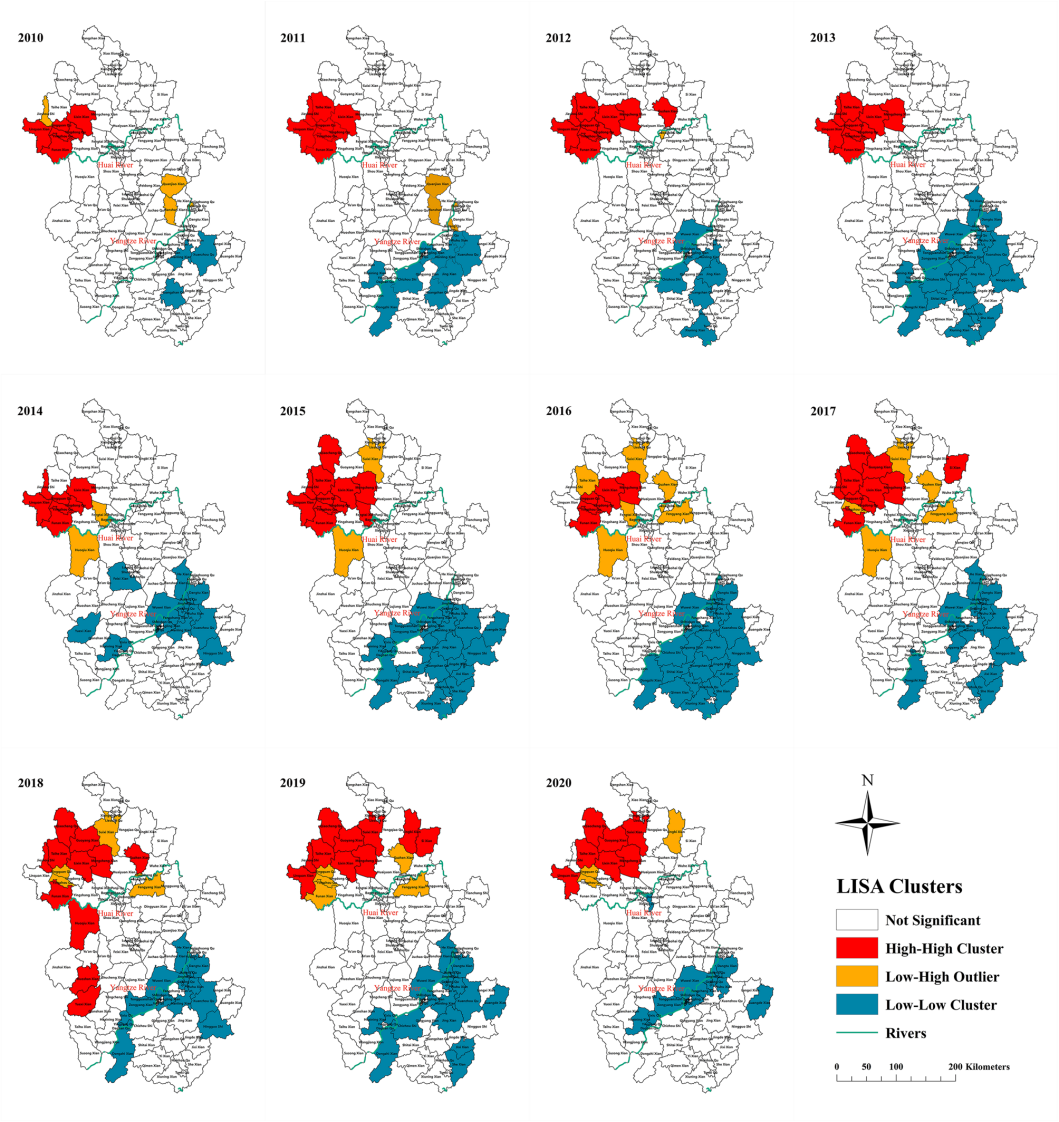
Yearly LISA cluster maps for scrub typhus incidence in Anhui, China, 2010–20. LISA spatial cluster map shows the center of the cluster in color. H-H indicates a statistically significant cluster of high scrub typhus incidence values; L-H represents low scrub typhus incidence values surrounded with high incidence values; L-L represents low scrub typhus incidence values surrounded with low incidence values. The base layer of the map was obtained from Resource and Environment Science and Data Center (https://www.resdc.cn/Datalist1.aspx?FieldTyepID=7,1).

The Global Moran’s* I* highlighted that the occurrence of scrub typhus outbreaks in Anhui Province exhibited overall aggregation throughout the study period. The Z-test indicated that Moran’s* I* was statistically significant over the past 11 years (Table 2). The Clusters and Outliers for each year were identified through LISA analysis and a LISA map was drawn on the basis of the Z-test (*p* < 0.05) (Fig. 5). The LISA analysis confirmed that the High-High Clusters were originally more commonly distributed in the plain area where located in the north of the Huai River, but the High-High Clusters appeared in the south of Huai River in 2018, and the range of High-High Clusters continued to expand. The results of LISA analysis demonstrated that the proportion of cases, counties, population, and areas in High-High Clusters displayed an increasing trend (Table 3).

**Table 2:** Yearly spatial autocorrelation analysis on scrub typhus incidence in Anhui, China, 2010–2020.

Year	Moran’s *I*	Z-Score	p-value
Average	0.35	6.05	< 0.05
2010	0.15	2.87	< 0.05
2011	0.24	4.39	< 0.05
2012	0.44	7.63	< 0.05
2013	0.40	7.20	< 0.05
2014	0.22	4.67	< 0.05
2015	0.31	5.63	< 0.05
2016	0.22	3.74	< 0.05
2017	0.27	4.66	< 0.05
2018	0.27	4.63	< 0.05
2019	0.39	6.48	< 0.05
2020	0.30	5.17	< 0.05

**Table 3:** Descriptive statistics of spatial clusters detected by LISA analysis in Anhui, 2010–2020.

Clusters	Incidence*(1/100,000)	%Cases	%Counties	%Population	%Area
2010					
HH	3.29	43.03	5.71	10.72	5.56
LH	0.26	0.82	3.81	2.61	2.41
LL	0.00	0.00	2.86	2.57	4.02
2011					
HH	6.27	49.02	7.62	12.69	7.38
LH	0.00	0.00	3.81	2.12	2.12
LL	0.02	0.10	8.57	7.22	11.56
2012					
HH	4.98	52.75	9.52	15.45	10.03
LH	0.00	0.00	0.95	0.41	0.10
LL	0.03	0.11	6.67	5.73	9.18
2013					
HH	3.91	47.47	8.57	14.35	8.95
LH	0.00	0.00	0.00	0.00	0.00
LL	0.02	0.28	20.00	15.73	23.67
2014					
HH	7.45	32.68	6.67	10.33	6.03
LH	0.63	0.91	1.90	0.02	3.51
LL	0.14	0.84	17.14	13.86	14.97
2015					
HH	9.04	40.79	10.48	17.76	11.38
LH	1.20	1.20	1.90	3.93	4.46
LL	0.07	0.33	24.76	17.87	23.79
2016					
HH	6.31	14.44	6.67	9.03	5.84
LH	1.63	4.01	6.67	9.68	9.16
LL	0.08	0.33	23.81	15.85	24.14
2017					
HH	5.19	41.19	9.71	16.64	11.76
LH	0.80	2.75	5.83	7.24	7.39
LL	0.09	0.61	18.10	14.35	15.64
2018					
HH	4.04	38.45	10.48	17.28	16.40
LH	0.70	2.09	4.76	5.41	4.01
LL	0.20	1.66	17.14	15.31	14.06
2019					
HH	12.59	59.12	9.52	18.77	14.08
LH	2.11	3.26	4.76	6.18	4.71
LL	0.23	0.98	20.95	17.04	18.90
2020					
HH	6.72	43.86	6.67	15.46	10.69
LH	1.36	2.35	2.86	4.10	2.39
LL	0.05	0.28	16.19	14.18	9.29

Incidence*: annual average incidence, calculated with the yearly counts of scrub typhus cases as a numerator and population size at the end of each year as a denominator; HH: High-High, a statistically significant cluster of high scrub typhus incidence values; LH: Low–High, low scrub typhus incidence values surrounded with High scrub typhus incidence values; LL: Low-Low, low scrub typhus incidence values surrounded with low scrub typhus incidence values.

### Spatial–temporal cluster analysis

**Figure 6:**
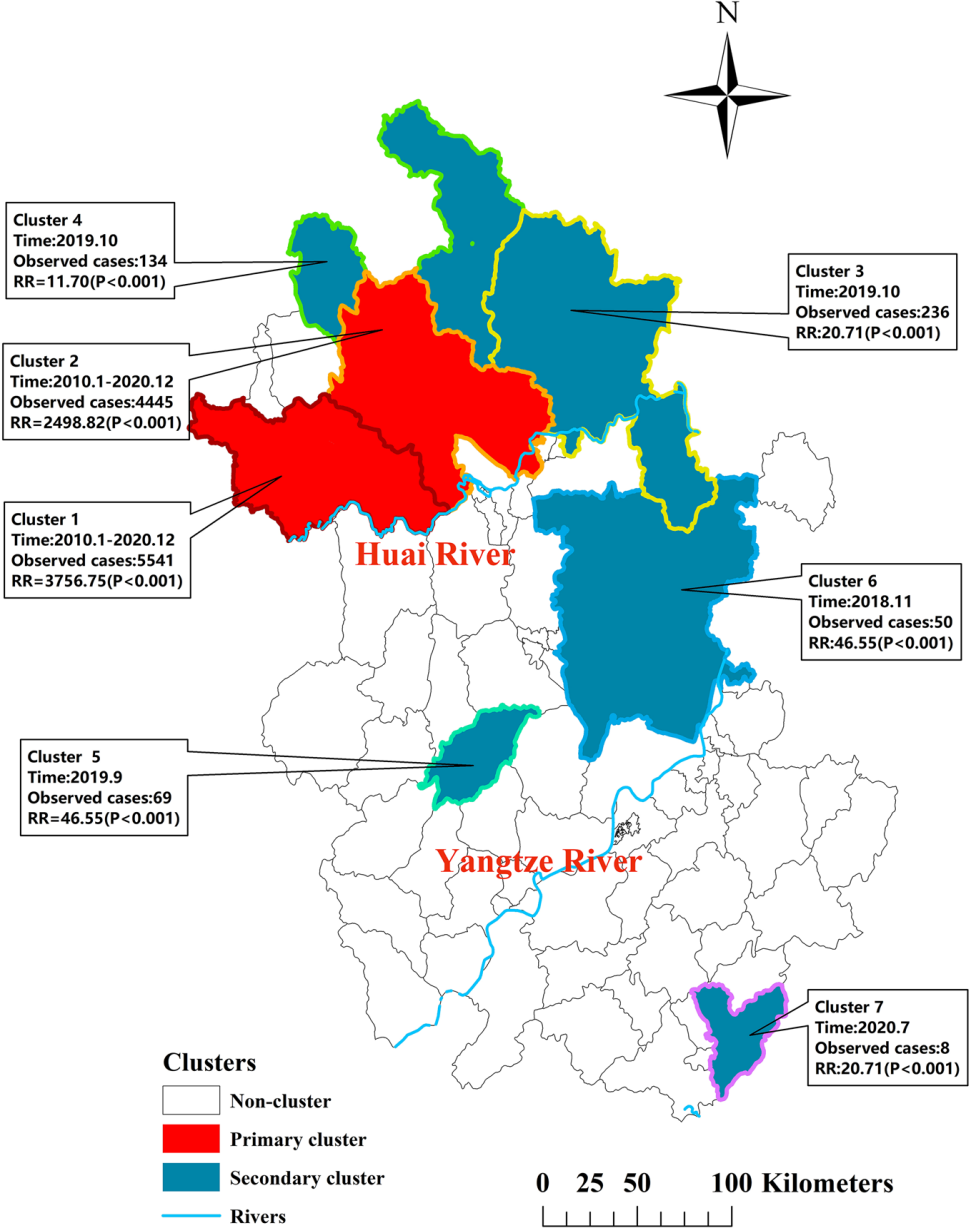
Spatiotemporal clusters of scrub typhus cases at the county level across the period of 2010–2020 in Anhui, China. The base layer of the map was obtained from Resource and Environment Science and Data Center (https://www.resdc.cn/Datalist1.aspx?FieldTyepID=7,1).

The result of Kulldorff’s space–time scan statistics was listed in Table 4. There were two significant clusters with *LLR* greater than 1000 identified in the 11-year period, which were defined as the primary clusters (Fig. 6). The primary clusters were located in the northwest part of Anhui Province, including 11 counties with 60.27% and 18.86% of the total number of cases and population respectively. Five significant secondary clusters detected in our study were dispersed throughout the northeastern, central-eastern, and southeastern parts of Anhui Province. The spatiotemporal distribution of scrub typhus in Anhui Province has undergone an extension since 2010.

**Table 4:** Spatiotemporal clusters of scrub typhus detected using Kulldoff’s space–time scan statistic in Anhui, China, 2010–2020.

Cluster	Lon	Lat	Radius (Km)	Time Frame	No. Counties	No Obs	No Exp	LLR	RR	Incidence (1/100,000)	% Population	% Cases
1※	115.64	32.65	57.35	2010/1–2020/12	6	5541	1526.28	2674.22	3756.75	93.04	9.92	33.44
2※	116.58	33.22	50.96	2010/1–2020/12	5	4445	1374.87	1444.10	2498.82	82.86	8.94	26.83
3^#^	117.88	33.54	84.93	2019/10	8	236	11.56	489.03	20.71	4.13	9.53	1.42
4	116.41	34.45	93.20	2019/10	7	134	11.55	206.52	11.70	2.38	9.38	0.81
5	116.82	31.31	0.00	2019/9	1	69	1.49	197.33	46.55	9.02	1.28	0.42
6	118.09	32.07	60.83	2018/11	11	50	8.97	44.92	5.59	1.09	7.64	0.30
7	118.57	29.81	0.00	2020/7	14	8	0.82	11.02	9.72	1.92	0.69	0.05

No. Counties, number of counties within clusters; No. Obs, number of observed cases; No. Exp, number of expected cases; LLR, log likelihood ratio; RR, relative risk of the cluster compared with the rest of the country; %, the proportion of the population or cases in cluster to the total during the clustering time.

Significant clusters with P < 0.05.

※1: Primary cluster.

^#^3–7: Secondary clusters.

### Association between the scrub typhus and environmental factors

The result of Pearson’s correlation analysis was listed in the Table 5. The variables EVP and TEM were excluded from negative binomial regression analysis due to their high correlation with variable GST. Considering that *L*. *scutellare* mites spend most of their life cycle on the ground except for the larval stage^21,22^, we included ground surface temperature with 0–3 months lags (GSTs) as the variables in our multivariate regression models to measure the effect of temperature, rather than air temperature (TEMs). The results of the negative binomial regression analysis were shown in Table 6. The correlation between POP, GST_lag3, and the monthly number of cases in univariate and multivariate analysis is opposite and this phenomenon might be caused by the inability to account for the interaction between variables in the univariate regression analysis. Twenty-five variables were excluded from the final multivariate regression model during the processing of preliminary Pearson’s correlation analysis and stepwise regression analysis. Each 1 ℃ increase of GST_lag1 was associated with a 14% (95%CI: 11–16%) increase in the number of cases in the current month, while a 1 mm increase of EVP_lag1 corresponded to a 54% (95%CI: 34–68%) decrease in the number of cases in the current month. The correspondence between other variables and the number of cases in the multivariate regression model were all listed in Table 6.

**Table 5:** Pearson’s correlation coefficient(‘r’)matrix of meteorological variables in Anhui, China, 2010–2020.

	EVP	WIN	GST	PRE	PRS	RHU	SSD	TEM
EVP	1.00							
WIN	0.10 (*p* = 0.00)	1.00						
GST	0.82 (*p* = 0.00)*	0.06 (*p* = 0.02)	1.00					
PRE	0.23 (*p* = 0.00)	0.17 (*p* = 0.00)	0.40 (*p* = 0.00)	1.00				
PRS	− 0.52 (*p* = 0.00)	− 0.26 (*p* = 0.00)	− 0.66 (*p* = 0.00)	− 0.44 (*p* = 0.00)	1.00			
RHU	− 0.08 (*p* = 0.00)	0.22 (*p* = 0.00)	0.19 (*p* = 0.00)	0.48 (*p* = 0.00)	− 0.2 2(*p* = 0.00)	1.00		
SSD	0.62 (*p* = 0.00)	− 0.02 (*p* = 0.48)	0.45 (*p* = 0.00)	− 0.19 (*p* = 0.00)	− 0.2 4(*p* = 0.00)	− 0.57 (*p* = 0.00)	1.00	
TEM	0.81 (*p* = 0.00)*	− 0.04 (*p* = 0.11)	0.99 (*p* = 0.00)*	0.43 (*p* = 0.00)	− 0.64 (*p* = 0.00)	0.21 (*p* = 0.00)	0.42 (*p* = 0.00)	1.00

*The variables with a threshold of Pearson’s correlation |r|> 0.7.

**Table 6:** The association between monthly scrub typhus cases and potential influencing factors by panel negative binomial regression.

Variables(Unit)	Univariate analysis		Multivariate analysis	
	Crude IRR (95%CI)	*p-*value	Adjusted IRR (95%CI)	*p-*value
POP(1000p/km^2^)*	0.67 (0.63, 0.72)	< 0.00	1.13 (1.03, 1.24)	< 0.00
WIN_lag0 (m/s)	0.10 (0.07, 0.15)	< 0.00	NS (excluded)	
WIN_lag1(m/s)	0.04 (0.03, 0.05)	< 0.00	NS (excluded)	
WIN_lag2 (m/s)	0.10 (0.08, 0.13)	< 0.00	0.46 (0.32, 0.66)	< 0.00
WIN_lag3 (m/s)	0.10 (0.07, 0.15)	< 0.00	0.47 (0.31, 0.70)	< 0.00
GST_lag0 (℃)	0.95 (0.93, 0.96)	< 0.00	NS (excluded)	
GST_lag1 (℃)	1.04 (1.02, 1.05)	< 0.00	1.14(1.11, 1.16)	< 0.00
GST_lag2 (℃)	1.09 (1.18, 1.10)	< 0.00	NS (excluded)	
GST_lag3 (℃)*	1.08 (1.07, 1.09)	< 0.00	0.95 (0.94, 0.97)	< 0.00
PRE_lag0 (dm)	0.99 (0.99, 1.00)	< 0.00	NS (excluded)	
PPE_lag1 (dm)	0.99 (0.99, 0.99)	< 0.00	NS (excluded)	
PRE_lag2 (dm)	1.00 (1.00, 1.00)	0.40	NS (excluded)	
PRE_lag3 (dm)	1.00 (1.00, 1.00)	< 0.00	NS (excluded)	
PRS_lag0 (hPa)	1.07 (1.06, 1.08)	< 0.00	NS (excluded)	
PRS_lag1 (hPa)	1.05 (1.05, 1.06)	< 0.00	1.03 (1.02, 1.04)	< 0.00
PRS_lag2 (hPa)	1.02 (1.00, 1.03)	< 0.00	NS (excluded)	
PRS_lag3 (hPa)	0.96(0.94, 0.97)	< 0.00	NS (excluded)	
RHU_lag0 (1%)	0.98 (0.97, 0.99)	< 0.00	0.95 (0.94, 0.96)	< 0.00
RHU_lag1 (1%)	0.99 (0.98, 1.00)	0.38	NS (excluded)	
RHU_lag2 (1%)	1.03 (1.02, 1.04)	< 0.00	NS (excluded)	
RHU_lag3 (1%)	1.04 (1.03, 1.06)	< 0.00	1.06 (1.05, 1.08)	< 0.00
SSD_lag0 (h)	0.86 (0.81, 0.91)	< 0.00	NS (excluded)	
SSD_lag1 (h)	0.93 (0.89, 0.98)	< 0.00	0.87 (0.82, 0.91)	< 0.00
SSD_lag2 (h)	1.16 (1.10, 1.22)	< 0.00	NS (excluded)	
SSD_lag3 (h)	1.28 (1.22, 1.35)	< 0.00	1.39 (1.28, 1.51)	< 0.00
NDVI^**#**^	1.07 (1.06, 1.08)	< 0.00	1.09 (1.07, 1.11)	< 0.00
Elevation (m)	0.99 (0.99, 0.99)	< 0.00	NS (excluded)	
Slop(°)	0.85 (0.84, 0.87)	< 0.00	0.92 (0.89, 0.94)	< 0.00
GDP(100,000RMB)	0.99 (0.99, 0.99)	< 0.00	NS (excluded)	

*The variables showed opposite correlations in univariate and multivariate analyses.

NS: Non-significant variables with p-value greater than 0.01.

NDVI^#^: Its value is equal to NDVI multiplied by 100; POP: Population density.

Factor_lag(k): factor with k-month lag, for example, EVP_lag1 represents EVP with 1-month lag.

## Discussion

The overall increase in the incidence could be partly attributed to the expansion of the epidemic area nationwide, the application of improved surveillance mechanisms, and the improvement of diagnostic in recent years^1^. The result that farmers, females, and the elderly were more prone to infection in Anhui Province was consistent with the results of other studies in China^6,15^. The high incidence among females and the elderly might be due to a large number of young people and middle-aged males in rural areas migrating to metropolises to live or work, leaving their older or female family members in their rural hometowns^11^. Moreover, with the implementation of China’s policies for ecosystem services, the damaged forests and wetlands recovered to some extent and the traditional burning of straw within rural areas was banned, which produced a favorable setting for the breeding of rodents and mites, increasing the risk of older and female family members infection while performing routine farming activities^23^.

Our study identified a unimodal season pattern peaking in October–November in Anhui and it can be well explained by the seasonal fluctuation of the population of the *L. scutellare* mites, which is the dominant vector mite in Anhui^24^. It was also found that *L. scutellare* mites began to appear in September, reached their peak in October, and began to decline in November, which was consistent with the seasonal distribution of the cases of scrub typhus^25^.

Interestingly, unlike other provinces where high-risk areas were located in the mountainous hilly regions, high-risk areas in Anhui Province were concentrated in the plain regions. Such difference in spatial distribution could be explained by two reasons. First, the lack of disease in the hilly and mountainous areas south of the Huai River in Anhui may just reflect the absence of the pathogen rather than the lack of a suitable environment for vectors^20^. Second, the high-incidence areas north of Huai River mainly plant wheat, soybean and cotton as the main crops^26^, and most of the soybean, and cotton in these areas are still harvested by traditional manual labor, which increased the risk of farmers being exposed to pathogen-carrying chigger mites.

Meteorological factors such as temperature, relative humidity, sunshine duration, and wind speed have been proven to have significant associations with the transmission and occurrence of scrub typhus^14,18,27^. The vectors of scrub typhus are ectothermic insects whose activity can be greatly affected by temperature. Therefore, the significant correlation between GSTs and scrub typhus is not surprising^27^. Since chiggers mainly reside in rodents, their spread is influenced by the activity area of the rodents^28^. The results of GST_lag1 in multivariate regression might confirm that high surface temperature favors host rodents’ activity. RHUs were thought to contribute to the viability of *L*. *scutellare* mites by giving proper humidity^14,16,18^, whereas, there is a negative correlation between RHU_lag0 and the occurrence of scrub typhus in multivariate regression, which differs from the expectation that the risk would be higher in wet environments. A possible explanation could be that fewer people and farmers tend to engage in outdoor activities and farm work when RHU is higher, which decreases the chances of people being exposed to mites^29^. The life cycle of* L*. *scutellare* mites includes seven stages (egg, prelarva, larva(chigger), nymphochrysalis, nymph, tritonymph and adult), of which only larva chiggers are parasitic^22^, so the successful attachment to the host is the key to the survival of the larvae^21^. Strong wind might not be conducive to the attachment of larvae to the host. Therefore, the WINs have a negative correlation with the scrub typhus occurrence in our study, and further studies are needed on the infection mechanism with wind speed.

The meteorological variables with 2 and 3 months lag seem to be related to the spawning condition of *L*. *scutellare* mites. For example, the adult trombiculidae stop their spawning when humidity is insufficient^27^. The result of current study also showed that RHU_lag3 was positively associated with scrub typhus in Anhui, each 1% rise in RHU_lag3 corresponded to an increase of 6% in the monthly number of scrub typhus cases. This finding is in general agreement with other studies^16–18^. The suitable spawning temperature for female mites is between 23 and 25 ℃^14^, but the GST in Anhui was above 30 ℃ during July–September. Hence, the negative correlation between GST_lag3 and scrub typhus cases might reflect that too high GST is not conducive to the oviposition and activity of mites. In contrast, RHU_lag3 and SSD_lag3 might provide a favored humidity and sunshine condition for fertilization, respectively.

The transmission of vector-borne diseases requires the host, while the existence and activity of the host might be affected by topographic conditions^29^. Elevation and slope have been included in our study as variables reflecting topographic conditions. The negative association between slope and scrub typhus might reflect that high slope is detrimental to host activity, while the regularity is not yet understood. This association might be explained by an increasing trend of cropland with decreasing slope gradients^30^.

The occurrence of scrub typhus was rarely reported in urban regions with lower vegetation cover than in rural regions^31^. The positive association between scrub typhus and NDVI provided support for this pattern^22^. Besides, the population density was added to the final multivariate regression model as a variable to remove the bias caused by the differences in population density across counties. Our result suggested that counties with a higher risk of scrub typhus had a higher population density. To assess the potential impact of urbanization level on scrub typhus, gross domestic product (GDP) was used as its proxy. The insignificant result of GDP might reflect that the economic level among high-incidence counties in Anhui was not very different.

Certainly, the limitations of this study should be acknowledged. First, all cases data used in our study were collected from a passive disease surveillance system, which inevitably suffers from underreporting. Data underreporting may lead to system deviation. However, since underreporting may have been consistent over the study period, it is reasonable to believe that the impact of the deviation is relatively minor^16^. Secondly, although we emphasized the potential variables that might influence its spatial distribution, we could not rule out confounding variables in our analyses, such as vector factors, host susceptibility, and more socio-economic variables other than GDP^32^. In addition, the underlying mechanistic links between scrub typhus and its related factors remain unclear. Finally, data on the accurate distribution of *L*. *scutell* are mites density and *O. tsutsugamushi* strains were unavailable, making it impossible to comprehensively present the picture of scrub typhus in Anhui involving cases, pathogens, hosts, and vectors^11^.

## Conclusion

This study is one of the most detailed studies on spatio-temporal epidemiology and the potential risk factors of scrub typhus in Anhui Province. The elderly and farmers were the most affected population groups and that scrub typhus has been increasing rapidly and widespread in Anhui Province. A comprehensive control strategy including publicity and public health education, rodent control, and provision of various protective equipment should be implemented in a targeted manner in endemic areas of Anhui Province. Given the complex nature of scrub typhus, further research requires more advanced machine learning and deep learning algorithms to explore more deeply the role of environmental and anthropological factors in the occurrence of scrub typhus and to predict the risk of scrub typhus accurately.

## Supplementary Information

Supplementary Information.

## Data Availability

Patient data are protected by the China CDC and are unsuitable for public sharing. Interested parties can apply for the data by contacting the data-center of China public health science (https://www.phsciencedata.cn/Share) or email data@chinacdc.cn. The base layer of the map was obtained from Resource and Environment Science and Data Center (https://www.resdc.cn/Datalist1.aspx?FieldTyepID=7,1).
