# Supplementary material for: Spatiotemporal dynamics and environmental determinants of scrub typhus in Anhui Province, China, 2010–2020
Source: Sci Rep. 2023 Feb 6;13:2131. doi: 10.1038/s41598-023-29373-7 (PMC9902522; doi:10.1038/s41598-023-29373-7)
Supplement: Supplementary file 1 — Supplementary Information. [file 41598_2023_29373_MOESM1_ESM.pdf]

Appendix Table 1. Summary of diagnosis criteria and classification for scrub typhus, mainland China Variable

	1952–2008	2009–present
Criteria or guidelines	Educational Book on Infectious Diseases (First–eighth edition)	National Scrub Typhus Control and Prevention Guideline. 2009
Issued by	Beijing: People's Medical Publishing House	Chinese Center for Disease Control and Prevention
Epidemiology linkage	1.1 An individual who experienced possible outdoor exposure to mite bites three weeks before the onset of illness, i.e., farming, fishing, camping, and straw collection, during the epidemic season of the disease	
Clinical description	2.1 Sudden high fever accompanied by characteristic eschar or ulcer.<br>2.2 Enlarged lymph nodes, skin rash, splenomegaly, or hepatomegaly.	2.1 Fever<br>2.2 Lymphadenopathy<br>2.3 Skin rash<br>2.4 Specific eschars/ulcers
Laboratory tests	3.1 An agglutination titer ≥1:160 in the Weil-Felix test using the OXK strain of *Proteus mirabilis*.<br>3.2 Seroconversion or a four-fold or greater rise in serum IgG antibody titers between acute and convalescent sera detected by using mixed Gilliam,Karp, Kato, and Kawasaki strains of *O. tsutsugamushi* as diagnostic antigen in indirect immunofluorescence antibody assay (IFA).<br>3.3 The detection of O. *tsutsugamushi* 56-kDa gene by polymerase chain reaction in clinical specimens.<br>3.4 The isolation of O. *tsutsugamushi* from clinical specimens.	
Diagnosis and classification	Probable or confirmed cases: a patient with any 3 items among 1.1, 2.1, 2.2, 3.1, 3.2.<br>The case was not further classified as probable or confirmed cases.	Suspected case:<br>(1) a patient with item 1.1, 2.1, plus either 2.2 or 2.3, and was excluded from other diseases. Or<br>(2) a patient with item 2.1, 2.2 and 2.3 during the local epidemic season of scrub typhus (May–November south of Yangtze River and October–November in north of Yangtze River).<br>Probable case:<br>(1) a suspected case with item 2.4. or<br>(2) a patient with item of 1.1, 2.1, and 2.4.<br>Confirmed case:<br>(1) a probable case with any one of the item 3.1–3.4. or<br>(2) a suspected case with any one of the item 3.2–3.4

Appendix Table 2. Detailed grouping of cases by common occupations

Occupations \ Year	2010	2011	2012	2013	2014	2015	2016	2017	2018	2019	2020
Farmer	491	860	834	690	1610	2114	2257	1202	1026	2397	1381
Household staff	2	13	12	11	25	68	52	23	23	45	28
Student	13	17	25	10	20	49	28	15	27	26	25
Pupils	25	40	65	22	54	78	54	24	11	27	17
Retiree	10	9	6	10	11	29	21	16	21	32	19
Teacher	7	8	9	5	10	16	19	10	11	12	3
Workers	22	25	16	7	17	45	30	19	22	39	18
Others	4	12	21	22	17	25	10	14	18	30	5

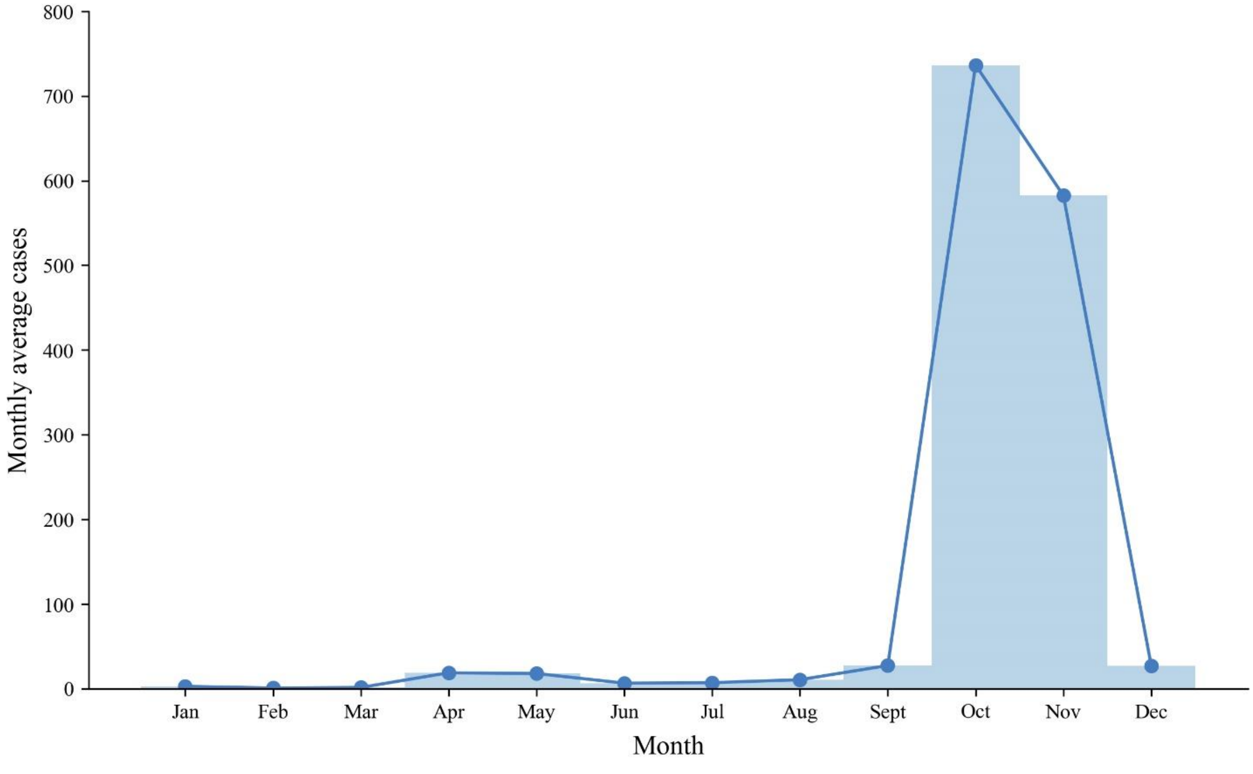

Appendix Figure 1. Monthly average number of cases per month from 2010 to 2020.
